# QTL mapping for soybean (*Glycine max* L.) leaf chlorophyll-content traits in a genotyped RIL population by using RAD-seq based high-density linkage map

**DOI:** 10.1186/s12864-020-07150-4

**Published:** 2020-10-23

**Authors:** Liang Wang, Brima Conteh, Linzhi Fang, Qiuju Xia, Hai Nian

**Affiliations:** 1 The State Key Laboratory for Conservation and Utilization of Subtropical Agro-bioresources, South China Agricultural University, Guangzhou, 510642 Guangdong People’s Republic of China; 2 The Key Laboratory of Plant Molecular Breeding of Guangdong Province, College of Agriculture, South China Agricultural University, Guangzhou, 510642 Guangdong People’s Republic of China; 3 Guangdong Provincial Laboratory of Lingnan Modern Agricultural Science and Technology, South China Agricultural University, Guangzhou, 510642 Guangdong People’s Republic of China; 4 Soybean Research Institute, National Center for Soybean Improvement, Key Laboratory of Biology and Genetic Improvement of Soybean (General, Ministry of Agriculture), State Key Laboratory of Crop Genetics and Germplasm Enhancement, Jiangsu Collaborative Innovation Center for Modern Crop Production, College of Agriculture, Nanjing Agricultural University, Nanjing, 210095 People’s Republic of China; 5 Beijing Genomics Institute (BGI) Education Center, University of Chinese Academy of Sciences, Shenzhen, 518083 Guangdong People’s Republic of China

**Keywords:** Soybean, Leaf chlorophyll-content traits, QTL mapping

## Abstract

**Background:**

Different soybean (*Glycine max* L.) leaf chlorophyll-content traits are considered to be significantly linked to soybean yield. To map the quantitative trait loci (QTLs) of soybean leaf chlorophyll-content traits, an advanced recombinant inbred line (RIL, ZH, Zhonghuang 24 × Huaxia 3) population was adopted to phenotypic data acquisitions for the target traits across six distinct environments (seasons and soybean growth stages). Moreover, the restriction site-associated DNA sequencing (RAD-seq) based high-density genetic linkage map of the RIL population was utilized for QTL mapping by carrying out the composite interval mapping (CIM) approach.

**Results:**

Correlation analyses showed that most traits were correlated with each other under specific chlorophyll assessing method and were regulated both by hereditary and environmental factors. In this study, 78 QTLs for soybean leaf chlorophyll-content traits were identified. Furthermore, 13 major QTLs and five important QTL hotspots were classified and highlighted from the detected QTLs. Finally, *Glyma01g15506*, *Glyma02g08910*, *Glyma02g11110*, *Glyma07g15960*, *Glyma15g19670* and *Glyma15g19810* were predicted from the genetic intervals of the major QTLs and important QTL hotspots.

**Conclusions:**

The detected QTLs and candidate genes may facilitate to gain a better understanding of the hereditary basis of soybean leaf chlorophyll-content traits and may be valuable to pave the way for the marker-assisted selection (MAS) breeding of the target traits.

**Supplementary information:**

**Supplementary information** accompanies this paper at 10.1186/s12864-020-07150-4.

## Background

Leaf chlorophylls are the major components in chloroplasts that affect photosynthetic capacities. The leaf chlorophylls were usually evaluated by using the extracted-based methods and can also be assessed by non-destructive approaches [1, 2]. Quantification by extract-based methods often involved the collections of leaf samples, solvent-based pigment extractions, and measurements of different chlorophylls (mainly chlorophyll a and chlorophyll b) by spectrophotometry [2–4]. Compared to the extraction methods, the soil plant analysis development (SPAD) assessing method by a handheld SPAD chlorophyll meter is non-destructive, fast and cheap for relative chlorophylls assessments [5]. Early studies have turned out that the soybean (*Glycine max* L.) leaf chlorophyll contents were positively correlated to seed yield during soybean reproductive stages [6, 7]. Hence, a better understanding of the genetic basis of chlorophyll-content traits in soybean leaves may be valuable for accelerating the soybean high-yield breeding.

Soybean leaf chlorophyll-contents were complex quantity traits controlled by polygenes that difficult to identify the major quantitative trait loci (QTLs) or functional genes [8]. Recently, the bottlenecks of traditional QTL mapping markers, like low genome coverage and uneven distributions, have been overcome by the rise of single nucleotide polymorphism (SNP) markers [9, 10]. Based on their genome-wide stability and abundance, SNP markers revolutionarily affected the high-density genetic map constructions and were adopted for seeking detailed hereditary information [11, 12]. To match with the massive DNA sequencing platforms, whole-genome sequencing (WGS) strategies have become the popular choices for next-generation sequencing (NGS) and were universally applied in SNP discovering and large population genotyping [13, 14]. These approaches mainly include resequencing, genotyping by sequencing (GBS) [15], specific length amplified fragment sequencing (SLAF-seq) [16], restriction site-associated DNA tag sequencing (RAD-seq) [17], and 2b-RAD [18]. The NGS technologies have been widely adopted in soybean, rice, wheat, sunflower and other crops [19–23]. Notably, QTL mapping by using soybean recombinant inbred line (RIL) populations and their RAD-seq based recombination bin marker genetic maps were reported to identify important soybean traits QTLs in recent years [24, 25]. Besides, the genome-wide association study (GWAS) was another primary mapping approach that relied on linkage disequilibrium genotyping arrays and investigated the associations of the SNPs throughout the entire genomes [26].

To date, there were 18 bi-parental and 6 GWAS QTLs for soybean leaf SPAD on SoyBase (https://www.soybase.org/) throughout different genetic backgrounds, sampling locations and mapping strategies. In another study, Dhanapal et al. collected 332 soybean varieties and assessed the chlorophyll a (eChl_A), chlorophyll b (eChl_B) and chlorophyll a/b ratio (eChl_R) in the soybean leaf extracts [2]. By carrying out the GWAS method, 14, 7 and 10 QTLs were identified for eChl_A, eChl_B and eChl_R, respectively [2]. In general, the QTLs for soybean leaf chlorophyll-content traits are still lacking compared to other soybean traits. Moreover, due to technical limitations, most bi-parental QTLs were detected by the traditional molecular markers and relatively low-density genetic maps.

In this study, we took the SPAD values as the indicators of the relative soybean leaf chlorophyll contents. These values were collected from the top, middle and bottom leaf sampling sites of the target soybean plants (TSP, top leaves SPAD value; MSP, mid leaves SPAD value; BSP, bottom leaves SPAD value). The average SPAD value (ASP) was the mean of TSP, MSP and BSP. Furthermore, based on spectrophotometer assessing of the top site sampled-leaf extracts, we quantified the target leaf chlorophylls a and b (Chl_A and Chl_B) contents and obtained the ratios of chlorophyll a to b (chlorophyll A/B ratios, Chl_A/B). For the assessments of these target soybean leaf chlorophyll-content traits, an advanced soybean RIL population (ZH RIL population) was adopted for the phenotypic data acquisitions. And the ZH RIL population was originated from the hybridization between ‘ZhongHuang 24’ (maternal plant) and ‘Huaxia 3’ (paternal plant) by using a single seed descent (SSD) method derived from individual F_2_ plants [27]. ‘Zhonghuang 24’ is a variety from the Institute of Crop Sciences, Chinese Academy of Agricultural Sciences, which adapts to the Huang-Huai-Hai growing region. ‘Huaxia 3’ is a high-yield soybean cultivar from South China Agricultural University. Previously, Liu et al. well-genotyped this RIL population and constructed a high-density bin marker map for the population with the RAD-seq approach [24]. Importantly, based on the yield difference between the parent soybeans of the RIL population, they finely mapped diverse QTLs for different soybean yield-related traits [24]. In the current investigation, we consecutively collected the phenotypic data of the chlorophyll-content traits across different seasons and soybean reproductive growth stages. By using the identical RIL population and its RAD-seq based high-density bin marker map, we identified 78 QTLs for the target traits. Concomitantly, we explored the relations between the QTLs identified in the present research and the published QTLs in the previous studies covering soybean leaf chlorophyll-content traits as well as soybean yield-related traits. Finally, we predicted six candidate genes for the target soybean leaf chlorophyll-content traits. In summary, the findings in the current study may provide clues for future gene functional research on soybean leaf chlorophyll-content traits and may be useful for promoting MAS (mark-assisted selection) breeding of high-yield soybean varieties.

## Results

### Phenotypic analyses of soybean leaf content traits with two distinct assessing methods

**Fig. 1:**
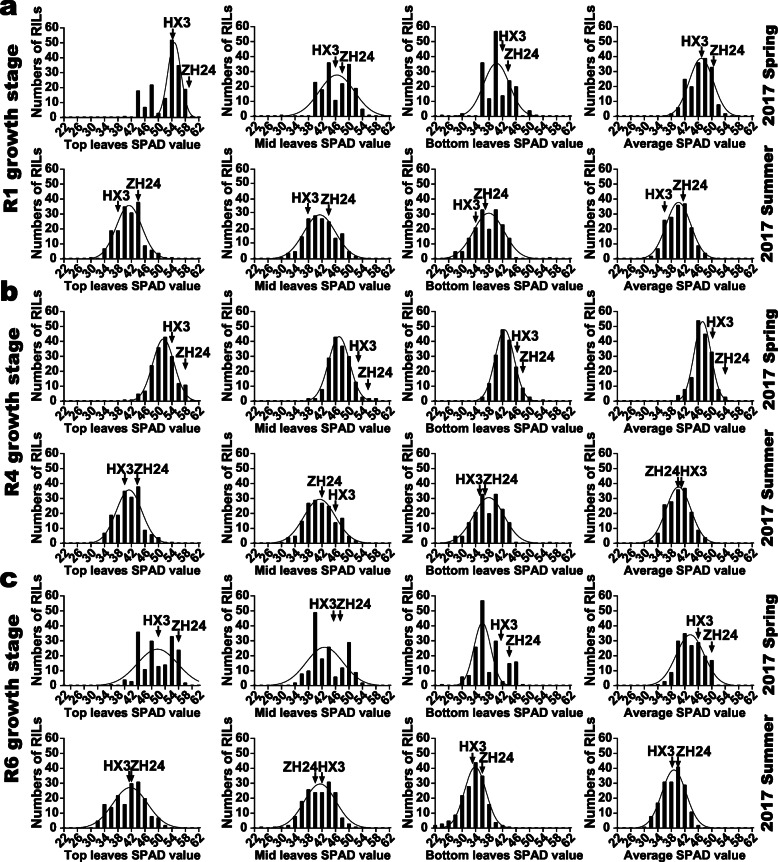
Frequency distributions for leaf SPAD-related traits in the ZH RILs. The arrows indicate traits associated values for the two parents used to construct the RIL population (cv. ‘Zhonghuang 24’ and ‘Huaxia 3’). **a** the frequency distributions for SPAD-related traits at soybean R1 growth stages in the spring and summer of 2017 at Zengcheng; **b** the frequency distributions for SPAD-related traits at soybean R4 growth stages in the spring and summer of 2017 at Zengcheng; **c** the frequency distributions for SPAD-related traits at soybean R6 growth stages in the spring and summer of 2017 at Zengcheng

**Fig. 2:**
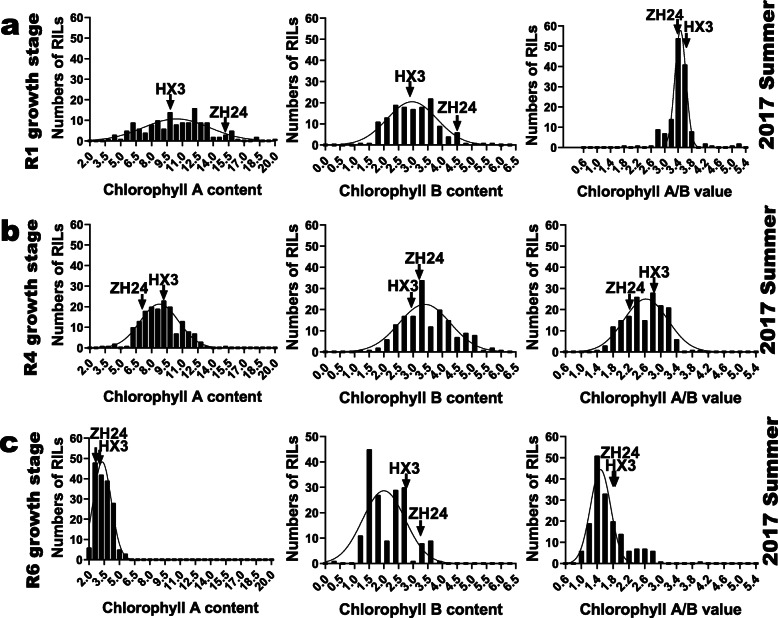
Frequency distributions for leaf extracting chlorophyll content traits in the ZH RILs. The arrows indicate traits associated values for the two parents used to construct the RIL population (cv. ‘Zhonghuang 24’ and ‘Huaxia 3’). **a** the frequency distributions for leaf extracting chlorophyll content traits at soybean R1 growth stage in the summer of 2017 at Zengcheng; **b** the frequency distributions for leaf extracting chlorophyll content traits at soybean R4 growth stage in the summer of 2017 at Zengcheng; **c** the frequency distributions for leaf chlorophylls content-related traits at soybean R6 growth stage in the summer of 2017 at Zengcheng

In previous studies, soybean leaf chlorophyll contents were reported to be positively correlated to seed yield during reproductive stages [6, 7]. Soybean leaf chlorophyll contents are complicated quantitative traits, which could be determined by different environmental and hereditary factors, such as, temperature and diverse growth stages of the RILs. To better map distinct QTLs for soybean leaf chlorophyll-content traits in the ZH RIL population, the phenotypic data and soybean leaf samples were collected at 9 a. m. according to the R1, R4 and R6 growth stages of different soybean lines across the spring and summer of 2017. Phenotypic data of TSP, MSP, BSP and ASP were collected across seasons and soybean growth stages. And Chl_A, Chl_B and Chl_A/B were phenotyped at different soybean growth stages. Most target traits of ‘Zhonghuang 24’ (maternal parent) presented higher values than those of ‘Huaxia 3’ (paternal parent), providing ideal materials for QTL mapping analyses. Generally, the phenotypic data of the RILs showed wide spans and displayed continuous distributions. Phenotypic frequency distributions of the target traits in the ZH RILs were depicted in Figs. 1 and 2. As is shown in the figures, the segregations of the chlorophyll-content traits fit normal or skew-normal distribution models, with typical quantitative genetic characteristics. Furthermore, the skewness and kurtosis of the distributions were listed in Table 1. Importantly, for each soybean leaf chlorophyll-content trait, the transgressive segregations widely presented in the RILs suggesting that the positive or negative alleles existed in the parent soybeans. Correlation analyses of the phenotypic data in RILs turned out that most chlorophyll-content traits were strongly correlated under the specific testing method (SPAD testing or spectrophotometer assessing of leaf extracts) and showed statistically significant (*P* < 0.01) (Additional file 1: Table S1). Interestingly, the SPAD values (TSP, BSP, MSP, and ASP) did not show significant correlations with Chl_A, Chl_B, or Chl_A/B in the current study. Overall, ASP presented the highest positive correlation coefficients to other SPAD traits. And Chl_A exhibited the strongest positive correlations to Chl_B and Chl_A/B. The Chl_A/B values were derived from the ratios Chl_A to Chl_B, which showed broadly positive correlations to Chl_A and negative correlations to Chl_B (with an exception of the R1 growth stage in the summer of 2017). Besides, we analyzed the phenotypic data correlations in different environments (Additional file 1: Table S2). As a result, the phenotypic data at the R6 growth stages in the spring and the summer of 2017 presented relatively better correlations with other circumstances.

**Table 1:** Leaf chlorophyll-content traits in the ZH RIL population across different environments

Traits	Parents Average		RILs Lines Average				Skewness	Kurtosis	Year, seasons and growth stages
	ZH24^a^	HX3^a^	Minimum^a^	Maximum^a^	Mean^a^	SD			
TSP	58.94 ± 0.82	54.58 ± 0.98	39.96	59.92	52.43	4.28	− 0.70	− 0.43	2017Spr-R1
MSP	48.17 ± 1.19	46.40 ± 1.27	29.50	55.64	46.35	4.20	−0.30	0.13	
BSP	43.37 ± 1.14	41.71 ± 1.64	24.58	51.60	40.55	4.09	−10.00	0.68	
ASP	50.16 ± 0.55	47.56 ± 0.59	37.30	54.90	46.46	3.31	−0.23	−0.43	
TSP	58.06 ± 0.52	53.57 ± 1.08	40.92	60.60	51.34	3.41	−0.16	0.16	2017Spr-R4
MSP	55.37 ± 1.43	52.82 ± 0.66	37.00	58.46	47.29	3.27	0.21	1.17	
BSP	47.06 ± 1.35	46.57 ± 1.51	35.34	53.60	42.87	2.90	0.27	0.74	
ASP	53.50 ± 0.44	50.98 ± 0.32	39.58	54.70	47.16	2.59	−0.23	0.42	
TSP	54.94 ± 1.24	50.40 ± 1.12	35.72	58.40	49.60	4.59	−0.24	− 0.85	2017Spr-R6
MSP	48.17 ± 0.66	46.40 ± 1.89	33.50	54.44	43.72	4.73	0.25	−0.93	
BSP	44.37 ± 1.22	41.71 ± 0.89	24.58	47.00	37.83	4.37	0.48	−0.14	
ASP	49.16 ± 0.59	46.17 ± 0.73	34.20	50.82	43.71	3.65	0.02	−0.60	
TSP	44.19 ± 1.54	37.89 ± 1.84	31.00	45.36	39.34	2.87	−0.30	−0.58	2017Sum-R1
MSP	42.90 ± 1.18	37.32 ± 0.92	29.66	45.84	38.07	3.22	−0.20	−0.26	
BSP	36.47 ± 1.09	33.57 ± 1.26	22.62	41.40	33.57	3.44	−0.55	0.42	
ASP	41.19 ± 0.62	36.26 ± 1.17	30.02	43.34	37.00	2.37	−0.29	0.27	
Chl_A^a^	15.12 ± 1.09	9.88 ± 0.60	3.84	20.26	10.39	3.12	0.26	0.04	
Chl_B^a^	4.49 ± 0.27	2.89 ± 0.26	1.17	5.79	3.01	0.82	0.52	0.43	
Chl_A/B	3.41 ± 0.30	3.55 ± 0.42	1.89	5.56	3.43	0.45	1.50	8.33	
TSP	42.39 ± 1.71	40.24 ± 1.02	30.90	54.22	41.24	3.81	0.25	0.54	2017Sum-R4
MSP	42.42 ± 0.96	46.23 ± 1.19	29.70	52.18	41.50	4.40	0.04	−0.37	
BSP	36.22 ± 1.25	35.89 ± 1.07	26.88	55.94	37.62	4.36	0.10	0.92	
ASP	40.34 ± 0.33	40.79 ± 0.54	30.84	48.14	40.12	3.43	−0.09	−0.38	
Chl_A^a^	7.04 ± 0.55	8.93 ± 0.36	2.97	15.29	8.92	1.95	0.12	0.50	
Chl_B^a^	3.24 ± 0.20	3.10 ± 0.07	1.22	6.70	3.55	0.96	0.54	0.50	
Chl_A/B	2.23 ± 0.28	2.88 ± 0.11	1.34	4.07	2.59	0.49	−0.09	−0.40	
TSP	41.85 ± 1.38	41.33 ± 0.73	31.00	52.94	41.21	4.60	0.00	−0.56	2017Sum-R6
MSP	40.46 ± 0.95	41.73 ± 1.23	23.56	52.80	41.02	4.42	−0.50	0.92	
BSP	35.72 ± 0.80	33.10 ± 0.85	22.24	44.30	32.72	3.75	−0.42	0.71	
ASP	39.34 ± 0.69	38.72 ± 0.81	29.58	46.30	38.51	3.26	−0.13	−0.55	
Chl_A^a^	5.17 ± 0.25	4.73 ± 0.31	2.17	5.65	3.38	0.77	0.35	−0.47	
Chl_B^a^	3.21 ± 0.40	2.83 ± 0.31	0.43	3.76	2.12	0.68	0.56	−0.31	
Chl_A/B	1.75 ± 0.30	1.76 ± 0.24	0.90	3.91	1.68	0.48	1.38	2.41	

*TSP* Top leaves SPAD value, *MSP* Mid leaves SPAD value, *BSP* Bottom leaves SPAD value, *ASP* Average SPAD value, *Chl_A* Chlorophyll a content, *Chl_B* Chlorophyll b content, *Chl_A/B* Chlorophyll a/b ratio, *ZH24* ‘Zhonghuang 24’, *HX3* ‘Huaxia 3’, ^*a*^ *mg/g,* 2017Spr: the spring of 2017, 2017Sum: the summer of 2017, *R1* R1 growth stage, *R4* R4 growth stage, *R6* R6 growth stage

### Mapping for the chlorophyll-content QTLs by using a genotyped high-density bin marker linkage map

**Fig. 3:**
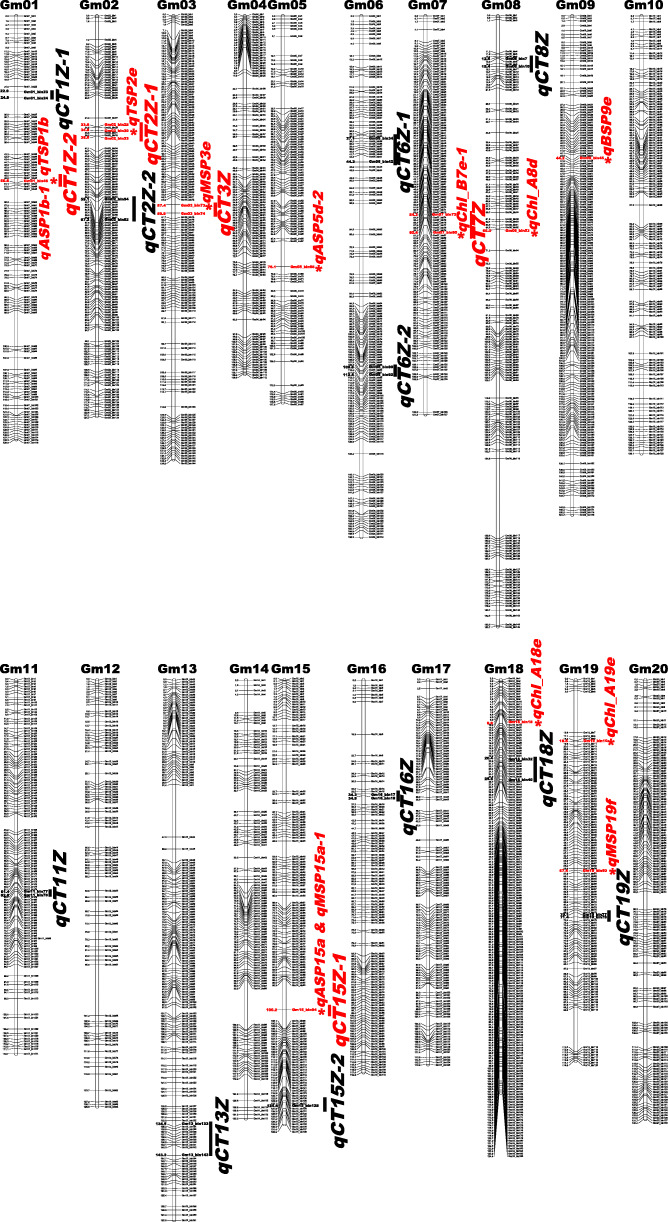
Soybean high-density genetic map of the ZH RIL population. Bin markers and their locations are shown on the right and left sides, respectively. The 16 chlorophyll-content traits QTL hotspots were depicted in bold and the 13 major QTLs were marked by asterisks in bold. Both the five important QTL hotspots and the 13 major QTLs were emphasized in red

The 47,472 high confidence polymorphic SNP sites were genotyped in the ZH RILs (Additional file 2: Fig. S1) and all the sites were then integrated as the recombination bin markers [24]. The SNP numbers in every bin marker on different chromosomes were listed in Table S3 (Additional file 1). And 2639 recombinant bin markers were acquired (Additional file 1: Table S4 and Additional file 3: Fig. S2). The bin physical length spanned from 20.01 kilobytes (kb) to 17.43 megabytes (Mb) with an average length of 360.01 kb. A high-density bin linkage map was constructed (Fig. 3), with an average distance of 1.00 cM between adjacent markers, covering 2638.24 cM in the *Glyma*.Wm82. a1. v1.1 reference genome. Chi-square test of the 2639 bin markers turned out that 2356 markers (89.28% of the total bin markers) displayed between parents with 1:1 segregation (*P* > 0.05), which was consistent with the characteristics of monogenic markers (Additional file 1: Table S5). Besides, 283 bin markers (account for 10.72%) exhibited separation distortion (*P* < 0.05). Most bin markers tended to be homozygous, and the heterozygous rate of bin markers was less than 4.79% (Additional file 1: Table S5).

By carrying out the composite interval mapping (CIM) method, 78 chlorophyll-content QTLs were identified distributing on the 19 soybean chromosomes (except for chromosome 20) (Additional file 1: Table S6). The phenotypic variations of the detected QTLs ranged from 5.10% (*qChl_B2f*) to 16.55% (*qASP1b-1*). There were 59 SPAD QTLs widely distributed on the 18 soybean chromosomes (except for chromosomes 12 and 20). Among these QTLs, 14, 18, 11 and 16 QTLs were mapped for TSP, MSP, BSP and ASP, respectively. *qTSP1b* and *qTSP2e* were the two dominant effect QTLs (*R*^*2*^ = 11.97 and 10.08%) for TSP. *qTSP1b* was identified by the bin46 marker on chromosome 01 and *qTSP2e* was detected by the bin30 marker on chromosome 02. Interestingly, *qASP1b-1* was also mapped by the bin46 marker on chromosome 01 and exhibited the highest phenotypic variance (*R*^*2*^ = 16.55%) among the identified QTLs. Besides, *qASP5d-2* and *qASP15a* (*R*^*2*^ = 12.05 and 11.94%) were designated as the other two major QTLs (*R*^*2*^ > 10%) for ASP. Coincidentally, *qMSP15a-1* fell in the identical physical interval with *qASP15a* and was one dominant effect QTL for MSP. Furthermore, *qMSP3e* and *qMSP19f* were the other two major MSP QTLs on chromosomes 03 and 15 (*R*^*2*^ = 10.06 and 15.50%). And *qBSP9e* was the only major QTL for BSP. In contrast, 19 extracting chlorophyll content QTLs were identified and distributed on the 12 soybean chromosomes with phenotypic variances spanned from 5.10% (*qChl_B2f*) to 13.55% (*qChl_A8d*). Three Chl_A major QTLs (*qChl_A8d*, *qChl_A18e* and *qChl_A19e*) and one Chl_B major QTL (*qChl_B7e-1*) were mapped on chromosomes 08, 18, 19 and 07, respectively. However, all the identified Chl_A/B QTLs were minor effect ones (*R*^*2*^ < 10%). Moreover, the 13 major QTLs (*qASP1b-1*, *qTSP1b*, *qTSP2e*, *qMSP3e*, *qASP5d-2*, *qChl_B7e-1*, *qChl_A8d*, *qBSP9e*, *qASP15a*, *qMSP15a-1*, *qChl_A18e*, *qChl_A19e* and *qMSP19f*) were highlighted in red and marked with asterisks in Fig. 3.

### Explorations of important bin markers and QTL hotspots for soybean leaf chlorophyll-content traits

As is shown in Table S6 (Additional file 1), five bin markers on four soybean chromosomes (01, 02, 07 and 15) were determined to be important and each of them identified QTLs for at least two distinct chlorophyll-content traits. Besides, bin30, bin80 and bin94 on chromosomes 02, 07 and 15 detected QTLs across different environments. In conclusion, the important bin markers may be valuable to detect the different chlorophyll-content QTLs throughout the environments.

According to the reported definition on QTL hotspots [24], 16 chlorophyll-content traits QTL hotspots on 12 soybean chromosomes (01, 02, 03, 06, 07, 08, 11, 13, 15, 16, 18 and 19) were summarized. These QTL hotspots contained at least two QTLs and were named after ‘CT’, which represented their regulations on different chlorophyll-content traits (Table 2). Besides, five important QTL hotspots (*qCT1Z-2*, *qCT2Z-1*, *qCT3Z*, *qCT7Z* and *qCT15Z-1*) were highlighted from the above QTL hotspots, and each of them contained at least one major QTL. As is shown in Table 2, *qCT1Z-2* consisted of two major SPAD QTLs (*qASP1b-1* and *qTSP1b*) that could explain the phenotypic variation ranging from 11.97 to 16.55%. And *qCT2Z-1* was anchored by three adjacent bin markers on chromosome 02 and contained four SPAD QTLs. Comparably, *qCT3Z* was in an interval from 36,603,803 to 37,138,700 bp on chromosome 03 and contained one QTL for MSP and one QTL for ASP. Furthermore, *qCT7Z* and *qCT15Z-1* are both important QTL hotspots, which included different SPAD and extracting chlorophyll content QTLs. And *qCT7Z* contained two extracting chlorophyll content QTLs and three SPAD QTLs and located in a block from 15,667,842 to 16,803,624 bp on chromosome 07. Moreover, *qCT15Z-1* consisted of two SPAD QTLs and one extracting chlorophyll content QTL. Notably, *qCT15Z-1* was anchored by the bin94 marker on chromosome 15 across distinct environments. The 16 chlorophyll-content traits QTL hotspots were marked in bold on the high-density genetic map. For the important five QTL hotspots, we further depicted them in red (Fig. 3).

**Table 2:** 16 QTL hotspots associated with leaf chlorophyll-content traits detected in the ZH RIL population

QTL hotspot^a^	QTL name^b^	Chr^c^	Bin name	CIv1.1 (bp)^d^	Bin length (bp)	Position (cM)	LOD^e^	ADD^f^	*R*^*2*^ (%)^g^	CIv2.0 (bp)^h^
*qCT1Z-1*	*qBSP1e*	1	Bin23	3,014,519-3,035,286	20,768	22.9	2.51	−1.08	5.81	3,037,245-3,038,559
	*qASP1f*	1	Bin24	3,035,287-3,300,327	265,041	24.8	4.02	−1.06	9.76	3,060,271-3,302,133
*qCT1Z-2*	*qASP1b-1*	1	Bin46	11,517,849-26,055,466	14,537,618	50.6	6.84	1.47	16.55	11,591,808-24,499,599
	*qTSP1b*	1	Bin46	11,517,849-26,055,466	14,537,618	50.6	5.13	1.66	11.97	11,591,808-24,499,599
*qCT2Z-1*	*qASP2e*	2	Bin28	6,826,912-6,946,505	119,594	33.6	2.60	−0.84	5.84	6,909,163-7,031,971
	*qBSP2d*	2	Bin30	7,060,106-7,152,841	92,736	34.8	3.66	−1.07	8.79	7,145,546-7,228,940
	*qTSP2e*	2	Bin30	7,060,106-7,152,841	92,736	34.8	4.23	−1.25	10.08	7,145,546-7,228,940
	*qBSP2f*	2	Bin33	7,454,452-9,601,062	2,146,611	38.6	2.77	−1.00	6.93	7,539,451-9,689,680
*qCT2Z-2*	*qChl_A/B2f*	2	Bin54	15,046,557-15,213,830	167,274	59.1	3.10	0.13	7.54	15,251,037-15,422,632
	*qChl_B2f*	2	Bin62	16,848,795-27,416,648	10,567,854	67.2	2.50	−0.16	5.10	17,128,244-26,385,905
*qCT3Z*	*qMSP3e*	3	Bin73	36,603,803-36,725,014	121,212	59.4	3.94	1.41	10.06	34,589,840-347,02,370
	*qASP3e*	3	Bin75	37,109,279-37,138,700	29,422	61.7	3.07	0.92	6.85	35,106,012-35,113,620
*qCT6Z-1*	*qASP6f*	6	Bin34	5,908,302-5,959,466	51,165	37.1	2.54	−0.80	6.07	5,914,573-5,964,561
	*qASP6d*	6	Bin42	6,959,298-7,246,025	286,728	44.2	3.92	−0.75	9.14	6,972,002-7,247,613
*qCT6Z-2*	*qTSP6b*	6	Bin89	15,950,387-16,194,517	244,131	109.8	3.10	0.93	7.45	16,011,413-16,229,680
	*qMSP6e-1*	6	Bin92	16,664,681-16,817,080	152,400	112.4	3.66	1.82	8.86	16,709,212-16,859,338
*qCT7Z*	*qMSP7a-1*	7	Bin73	15,667,842-15,734,307	66,466	54.3	3.23	−1.18	7.21	15,769,608-15,813,498
	*qASP7a*	7	Bin80	16,504,261-16,803,624	299,364	60.4	2.74	−0.84	6.07	16,597,814-16,889,324
	*qChl_B7e-1*	7	Bin80	16,504,261-16,803,624	299,364	60.4	5.05	−0.39	12.75	16,597,814-16,889,324
	*qChl_A/B7d*	7	Bin80	16,504,261-16,803,624	299,364	60.4	2.83	−0.18	8.69	16,597,814-16,889,324
	*qMSP7a-2*	7	Bin80	16,504,261-16,803,624	299,364	60.4	2.58	−1.06	5.81	16,597,814-16,889,324
*qCT8Z*	*qTSP8f-1*	8	Bin7	2,225,026-2,271,343	46,318	12.6	3.10	1.30	7.64	2,238,240-2,275,653
	*qMSP8b*	8	Bin10	2,712,995-2,737,327	24,333	15.4	2.80	0.98	7.05	2,719,028-2,741,745
*qCT11Z*	*qChl_A/B11e-2*	*11*	Bin77	16,152,967-16,449,587	296,621	61.4	2.91	0.14	7.16	26,145,820-26,431,037
	*qBSP11e*	*11*	Bin78	16,449,588-16,610,503	160,916	62.6	3.07	−1.18	7.10	26,412,450-26,574,724
*qCT13Z*	*qASP13b-1*	*13*	Bin133	39,361,093-39,386,817	25,725	134.5	2.74	−0.67	6.30	40,532,836-40,567,479
	*qASP13b-2*	*13*	Bin143	40,173,283-40,428,061	254,779	143.3	2.71	−0.66	6.25	41,618,514-41,874,899
*qCT15Z-1*	*qASP15a*	*15*	Bin94	16,683,420-17,383,420	700,001	100.2	5.27	−1.19	11.94	16,695,707-17,350,040
	*qChl_B15f-1*	*15*	Bin94	16,683,420-17,383,420	700,001	100.2	2.55	−0.17	5.71	16,695,707-17,350,040
	*qMSP15a-1*	*15*	Bin94	16,683,420-17,383,420	700,001	100.2	5.94	−1.63	13.86	16,695,707-17,350,040
*qCT15Z-2*	*qChl_A15f*	*15*	Bin128	48,582,583-48,741,529	158,947	124.4	3.68	0.23	8.99	49,382,502-49,534,743
	*qChl_B15f-2*	*15*	Bin128	48,582,583-48,741,529	158,947	124.4	3.97	0.20	8.94	49,382,502-49,534,743
*qCT16Z*	*qTSP16e*	*16*	Bin17	5,525,628-5,588,607	62,980	34.4	3.29	1.08	7.69	5,551,673-5,607,211
	*qASP16e*	*16*	Bin18	5,588,608-5,809,086	220,479	34.9	3.30	0.96	7.61	5,618,869-5,828,808
*qCT18Z*	*qMSP18f-1*	*18*	Bin32	3,157,973-3,350,322	192,350	20.4	3.41	1.24	7.68	3,182,661-3,349,093
	*qMSP18f-2*	*18*	Bin40	4,116,314-4,142,492	26,179	25.6	3.30	1.21	7.44	4,139,387-4,161,508
*qCT19Z*	*qTSP19f*	*19*	Bin77	40,662,371-40,701,058	38,688	70.1	3.28	1.33	8.17	40,859,609-40,891,331
	*qMSP19d*	*19*	Bin78	40,701,059-40,809,377	108,319	71.4	2.66	−0.85	6.57	40,896,924-41,007,822

^a^The name of the QTL hotspot is a composite of multiple chlorophyll-content traits (CT); Zhonghuang 24 × Huaxia 3 RIL population (Z)

^b^The name of the QTL is a composite of the chlorophyll-content traits: TSP: Top leaves SPAD value, MSP: Mid leaves SPAD value, BSP: Bottom leaves SPAD value, ASP: Average SPAD value, Chl_A: Chlorophyll a content, Chl_B: Chlorophyll b content, Chl_A/B: Chlorophyll a/b ratio; a: the R1 growth stage in the spring of 2017 at Zengcheng; b: the R4 growth stage in the spring of 2017 at Zengcheng; c: the R6 growth stage in the spring of 2017 at Zengcheng; d: the R1 growth stage in the summer of 2017 at Zengcheng; e: the R4 growth stage in the summer of 2017 at Zengcheng; f: the R6 growth stage in the summer of 2017 at Zengcheng

^c^Chr refers to the chromosome

^d^The physical position corresponding to the 95% confidence interval for the detected QTL based on *Glyma*.Wm82. a1. v1. 1 gene model

^e^LOD indicates the logarithm of the odds score

^f^Positive and negative values indicated additive effect by the alleles of ‘Zhonghuang 24’ and ‘Huaxia 3’, respectively

^g^*R*^*2*^ indicates the phenotypic variance explained by individual QTL

^h^The most proximal *Glyma*.Wm82. a2. v1 gene model physical intervals of the detected QTLs were transformed by focusing on the positions of the interval nearest 3′ and 5′ ending genes

### Relations between the identified QTLs in the present research and the previous studies

Previously, there were 24 published SPAD QTLs spanned on 12 soybean chromosomes on SoyBase (Additional file 1: Table S7) [28–31]. In this study, we analyzed the relations between the identified soybean leaf chlorophyll content traits QTLs and the published SPAD QTLs (Additional file 1: Table S8). As a result, 17 detected QTLs were associated with 11 reported QTLs. And most identified QTLs in the current research were novel ones for soybean leaf content traits.

**Fig. 4:**
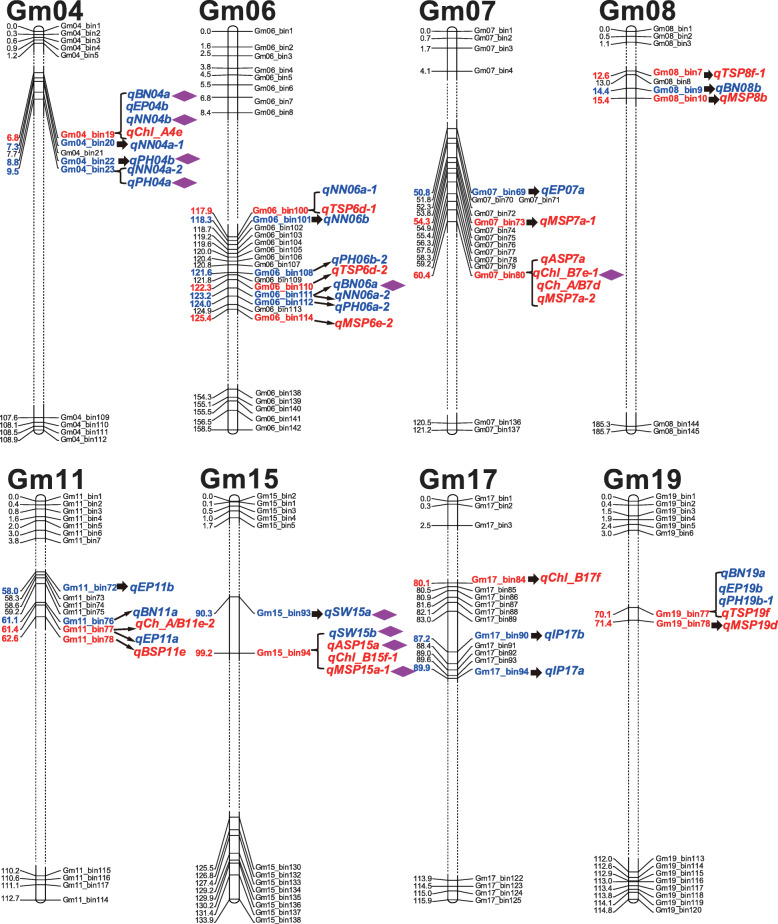
Relations between the identified QTLs and published yield-related QTLs in the identical genetic map. The virtual point lines represent the truncated segments of the chromosomes. The chlorophyll-content QTLs and the published yield-related QTLs were colored in red and blue, respectively [24]. The corresponding bin markers have been highlighted in bold. Both the major QTLs for chlorophyll-content traits and yield traits were emphasized and marked with the purple diamonds. Some abbreviations in formerly reported yield-related QTLs: BN, branch number; EP, effective pod; IP, invalid pod; NN, nod number; PH, plant height; SW, seed weight; a, in the summer of 2012; b, in the summer of 2015

Various yield-associated traits like lodging, plant height, seed set (also named seed per pod), seed weight, internode length, seed yield, could affect soybean yield [29, 32–45]. In a former study, Liu et al. utilized the same mapping RIL (the ZH RIL) population and elegantly identified QTLs for different yield and quality-related traits [24]. Based on the identical mapping population and the genetic map, we further explored the correlations between the chlorophyll-content QTLs in this study and the yield-related traits QTLs reported by Liu et al. [24]. As a result, 19 identified chlorophyll-content QTLs spanned on eight chromosomes (04, 06, 07, 08, 11, 15, 17 and 19) were found to be associated with the 25 published yield-related QTLs (Fig. 4). Concomitantly, both the detected chlorophyll-content traits major QTLs and the published yield-related traits major QTLs were highlighted with the purple diamond tags on the genetic linkage map. Besides, different recorded yield-related traits QTLs on SoyBase were also found in the genetic regions of the five important QTL hotspots in this study (Additional file 1: Table S9). In conclusion, the correlated chlorophyll-content and yield-related QTLs in the present and previous studies, especially the major QTLs, may be valuable to pave the way for soybean high-yielding breeding.

### Gene ontology (GO) enrichment analyses, candidate gene predictions, candidate gene genomic locations and gene structure characteristics

To gain an in-depth understanding of which genes may relate to soybean leaf chlorophyll-content traits, we retrieved the gene calls in the genetic blocks of the 13 major QTLs and the five important QTL hotspots. Totally 515 and 410 annotated genes were found in the corresponding hereditary intervals of the major QTLs and important QTL hotspots, respectively (Additional file 1: Table S10 and Table S11). Among the annotated genes, 384 were corresponded to at least one GO annotation. Moreover, these genes were predicted to be associated with various biological processes and could further classify into 21 categories, including signaling, regulation of biological process, biological regulation, cellular process, signaling process, metabolic process, localization, response to stimulus, transporter activity, transcription regulator activity, structural molecule activity, catalytic activity, binding, enzyme regulator activity, electron carrier activity, macromolecular complex, cell, cell part, organelle and organelle part (Additional file 1: Table S12). Some biological processes such as metabolic processes, catalytic activity, and especially transcription regulator activities are crucial for gene expression and metabolites in different organisms. Furthermore, the GO enrichment terms in Table S12 (Additional file 1) were filtered by *P* < 0.05 and gene number over five in Table S13 (Additional file 1). As a result, 17 GO terms in the molecular function class and eight GO terms in the biological process were filtered. And the filtered GO terms were depicted in Fig. S3 (Additional file 4).

**Fig. 5:**
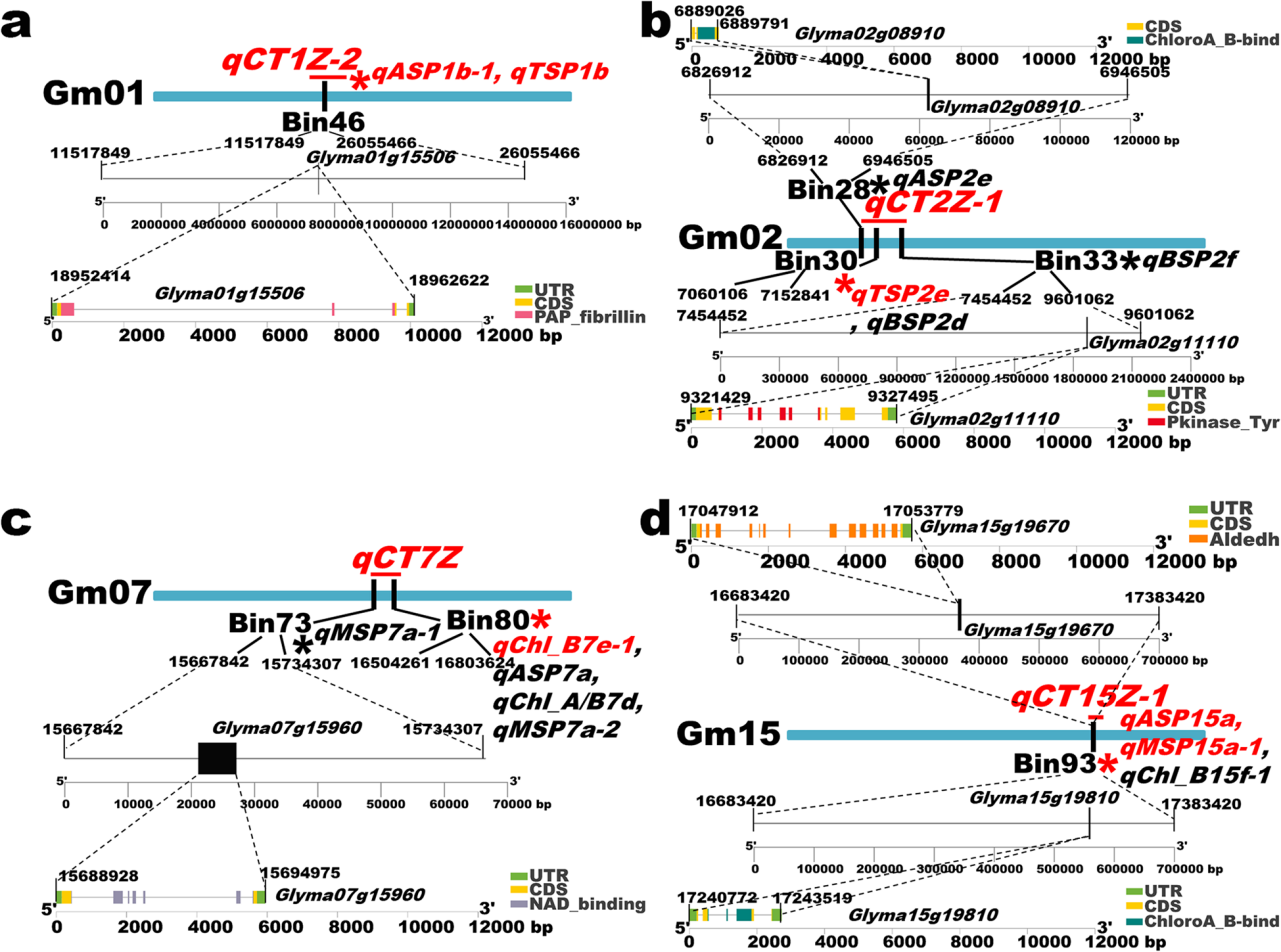
Candidate gene genomic locations, conserved domains and gene structures. **a** The genomic location, conserved domain and gene structure of *Glyma01g15506*; **b** The genomic locations, conserved domains and gene structures of *Glyma02g08910* and *Glyma02g11110*; **c** The genomic location, conserved domain and gene structure of *Glyma07g15960*; **d** The genomic locations, conserved domains and gene structures of *Glyma15g19670* and *Glyma15g19810*. The candidate genes were originated from the genetic intervals of distinct major QTL hotspots. The bin markers were corresponding to different chlorophyll-content traits QTLs in this study. Both the major QTL hotspots and major QTLs were emphasized in red color. The gray lines in gene structure diagrams indicate introns and the different gene structural components were exhibited by different colored boxes. UTR: untranslated region; CDS: coding sequence

Based on the GO enrichment analyses, the gene annotations on SoyBase (Additional file 1: Table S14) and the gene expression levels on SoyBase and Phytozome (https://phytozome.jgi.doe.gov/pz/portal.html), we predicted six candidate genes for soybean leaf chlorophyll-content traits. Among them, *Glyma01g15506* was in the genetic block of two major QTLs (*qTSP1b* and *qASP1b-1*) and the two major QTLs were identified by the same bin marker in *qCT1Z-2*. Moreover, *Glyma02g08910* and *Glyma02g11110* were originated from the different hereditary regions of *qCT2Z-1*. Furthermore, *Glyma07g15960* was predicted as a putative functional gene in *qCT7Z*. Besides, *Glyma15g19670* and *Glyma15g19810* were two adjacent genes obtained from the identical genetic interval of two major QTLs (*qASP15a* and *qMSP15a-1*) in *qCT15Z-1*. The detailed genomic locations, conserved domains and gene structures of the candidate genes were depicted in Fig. 5. The genomic positions of the candidate genes were stepwise displayed from the anchored bin markers on soybean chromosomes to the candidate genes in the bin markers. The bin markers and the candidate genes were displayed at the ratios based on the soybean genome physical locations. According to Fig. 5, *Glyma01g15506, Glyma02g08910*, *Glyma02g11110*, *Glyma07g15960, Glyma15g19670* and *Glyma15g19810* contained plastid-lipid associated protein (PAP) fibrillin domain, chlorophyll A-B binding (ChloroA_B-bind) domain, threonine-protein kinase (Pkinase_Tyr) domain, nicotinamide adenine dinucleotide (NAD) binding domain, aldehyde dehydrogenase (Aldedh) domain and chlorophyll A-B binding domain, respectively.

## Discussion

### Variations in soybean leaf chlorophyll-content traits

Chlorophylls are the major photosynthetic pigments acting as the main absorbers of harvesting light in plants [46, 47]. Soybean chlorophyll-content traits are complex quantitative traits involving in the dual-effects of hereditary and environmental factors [48, 49]. As is shown in Table 1, the phenotypic data of chlorophyll-content traits fluctuated with the soybean growth stages. For MSP, BSP and ASP, the phenotypic data dynamically increased from the R1 to R4 growth stages and then decreased from the R4 to R6 growth stages. Interestingly, phenotypic data of TSP, Chl_A, Chl_B and Chl_A/B gradually reduced with the three soybean reproductive growth stages. Furthermore, for the specific trait, the paternal soybeans generally showed lower values than those of maternal ones. Taken together, we speculated that soybean leaf chlorophyll-contents may be determined by the genetic differences in the RILs and are regulated by distinct functional genes during different growth stages [25, 50]. Moreover, according to the correlation analyses in Table S1 (Additional file 1), most chlorophyll-content traits were highly correlated under specific chlorophyll assessing methods. Hence, the regulating genes may be pleiotropic or closely linked [51]. Both the extracted-based or SPAD-based methods are popular approaches in chlorophyll assessments. However, the phenotypic data under distinct evaluating approaches show no obvious correlation, which may due to the different mechanisms and phenotyping focuses of the two chlorophyll assessing methods in this study. For the mechanisms, the SPAD testing method is a non-destructive way that measures light transmittance of the leaf in the red and infrared wavelengths at 650 and 940 nm to assess the relative total chlorophyll content in leaves [5]. Whereas, the extracted-based method is a destructive approach detecting by a spectrophotometer depending on different light wavelengths (645 and 663 nm) to assess different components of leaf chlorophylls (Chl_A and Chl_B) [52]. For the phenotyping focuses, SPAD testing in this study was focused on different leaves from distinct positions of soybean plants. Whereas, for the extracted-based method, we sampled the top position leaves (the fully developed middle leaflets of the 3rd node on the main stem counting down from the top) for soybean leaf pigment extractions. In most cases, the plants from different soybean lines of the RIL population exhibited diverse plant architectures. And the upper position leaves of the plant shaded more or less on the lower position ones throughout different soybean lines of the RIL population, which may affect the SPAD testing reads as well as the correlations between the two chlorophyll assessing methods. Besides, phenotypic data at the same soybean growth stages but different planting seasons exhibited variations, and this may highlight the effects of environmental factors on relevant traits. In all, variations of the soybean leaf chlorophyll-content traits support the reported characteristics of quantitative traits [29–31].

### Main effective factors for QTL mapping

QTL mapping is commonly used to identify genomic regions responsible for important quantitative and quality traits and has been regarded as a powerful tool for favorable alleles digging in the crop improvement process. Generally, QTL mapping depends on detecting the correlations between genetic markers and phenotypic traits in a segregating population [53, 54]. This study aimed to explore QTLs for soybean leaf chlorophyll-content traits. Many factors such as the types and the scales of the mapping populations, parental genetic backgrounds, hereditary capacities, genetic markers densities and distributions, environmental factors could affect the mapping precisions. Despite 18 bi-parental SPAD QTLs were recorded on SoyBase, however, the limitations of mapping populations and technologies restricted the discovering of important QTLs or genes. To avoid the former disadvantages, we improved them from different aspects, including the application of an F_12_ advanced RIL population with sufficient segregation lines (164 ZH RILs) and its RAD-seq based high-density genetic bin marker map. Hence, we gathered more accurate hereditary information for the target traits QTLs. Some chlorophyll-content traits QTLs were identified by the bin markers that spanned relatively large genetic intervals (Additional file 1: Table S6). For instance, *qASP1b-1* and *qTSP1b* were detected by the bin46 marker on chromosome 01. Although considerable SNPs (51 SNPs) existed in the bin marker (Additional file 1: Table S3), however, this bin marker spanned a large genetic interval (14,537,618 bp). One possible cause is that the bin marker may locate in a heterochromatic region, where the frequency of the recombination events is low [55]. The practicable strategy to further fine map these QTLs is to increase the mapping population size and to screen for the valuable recombinant lines [56]. Notably, the parental soybeans broadly exhibited differences in the chlorophyll-content traits (Table 1, Figs. 1 and 2). In most cases, ‘Zhong Huang 24’ (maternal soybean) took the surpassing values compared to those of ‘Huaxia 3’ (paternal soybean). Previous studies demonstrated that the significant positive correlations between seed yield and leaf chlorophyll content were usually found in soybean reproductive stages [7]. In this study, we focused on three representative important stages (R1, R4 and R6) throughout the soybean early, medium and late reproductive stages for the phenotypic data collections. Furthermore, we aggregately analyzed target traits with two chlorophyll valuating approaches (SPAD testing and leaf extracting assessing) in the spring and summer of 2017. Both two methods were widely adopted in leaf chlorophyll evaluations [2, 5]. The SPAD testing method is fast and convenient for relatively total leaf chlorophyll measurements. For the analyses of chlorophyll-related components, it is recommended to carry out the leaf extracting method [2, 5]. In conclusion, the detected QTLs in the current study expanded the QTLs for soybean leaf chlorophyll-content traits and may be beneficial to soybean MAS breeding for associated soybean leaf chlorophyll-content traits.

### Correlations between the identified chlorophyll-content traits QTLs and reported diverse traits QTLs

Compared to the genetic regions of recorded SPAD QTLs on SoyBase, most QTLs identified in this study were in novel hereditary regions. Due to relatively low density and uneven marker distributions of the former genetic linkage maps, these genetic regions may not be detected. Hence, our findings may facilitate to enrich the genetic information for soybean leaf chlorophyll-content traits QTLs.

Soybean is a crucial crop abundant with healthy protein and oil, and high yield is one of the most important targets in soybean breeding [25, 57]. Early studies turned out that the soybean leaf chlorophyll contents could be positively correlated to soybean yield [6, 7]. Previously, the ZH RIL population and the high-density bin marker map were adopted to detect distinct yield-related traits QTLs [24]. In this study, we carried out comprehensive comparisons between the identified chlorophyll-content traits QTLs and the published yield-related QTLs. The results were schematically presented in Fig. 4, which may reveal the potential relationships between the chlorophyll-contents QTLs and yield-related QTLs. For instance, the bin19 marker on soybean chromosome 04 identified a QTL for Chl_A in this study. And this bin marker was also associated with a reported QTL for the effective pod (EP) and two published major QTLs for branch number (BN) and nod number (NN) [24]. Moreover, the bin94 marker on chromosome 15 detected two major QTLs for ASP and MSP, and this bin marker was associated with reported QTLs for seed weight (SW). Besides, we found some recorded yield-related QTLs on SoyBase located in genetic intervals of the five important QTL hotspots for chlorophyll-content traits (Additional file 1: Table S9). This study focused on the QTL mapping of distinct soybean leaf chlorophyll-content traits and we also investigated the relations between the identified QTLs for soybean leaf chlorophyll-content traits in the current study and the early reported QTLs for soybean yield-related traits. However, future phenotyping and correlation analyses between the soybean leaf chlorophyll-content traits and relevant yield traits still need to be performed to further explore the underlying relations between leaf chlorophyll content and soybean yield.

### Six candidate genes for the chlorophyll-content traits

The leaf chlorophyll contents were determined by the dynamically biosynthesis and metabolism of chlorophylls in plant leaves [58]. Based on the gene annotations and expression levels of the identified genes, we predicted six candidate genes for chlorophyll-content traits in the hereditary intervals of the major QTLs and the QTL hotspots. In tomato, earlier research reported that fibrillin affected plastid ultrastructures and pigment contents [59]. In this study, *Glyma01g15506* was predicted to encode a plastid-lipid associated protein (PAP) in the fibrillin protein family, which was extracted from the identical genetic region of two major QTLs (*qTSP1b* and *qASP1b-1*) in *qCT1Z-2*. And this gene might affect the chlorophyll contents in soybean leaves. Another two putative genes, *Glyma02g08910* and *Glyma02g11110*, were derived from the hereditary intervals of *qCT2Z-1.* The nuclear *CAB* (chlorophyll A-B binding) gene families in plant encoded 20 to 24 kD proteins, which spanned chloroplast thylakoid membranes and coordinated distinct chlorophylls and carotenoids [60]. *Glyma02g08910* was responsible for a chlorophyll A-B binding protein, which was associated with the absorption and transfer of the energy absorbed from light photons between photosystem reaction centers [61]. And it could be regarded as one of the candidate genes that affecting soybean leaf chlorophylls. *Glyma02g11110* encoded a serine/threonine-protein kinase and was annotated to be associated with the chlorophyll catabolic process on SoyBase (Additional file 1: Table S14). In a previous study, Sun et al. overexpressed one wild soybean serine/threonine-protein kinase gene (*GsSRK*) in Arabidopsis. As a result, the transgenic Arabidopsis lines exhibited higher chlorophyll contents and improving salt tolerance abilities compared to those in wild type Arabidopsis [62]. Hence, we predicted *Glyma02g11110* as a candidate gene that may affect the chlorophyll contents in soybean leaves. Furthermore, *Glyma15g19670* and *Glyma15g19810* were two neighboring candidate genes, which were obtained from the same genetic regions of two major QTLs (*qASP15a* and *qMSP15a-1*) in *qCT15Z-1*. Coincidently, *Glyma15g19810* was the candidate gene for soybean leaf chlorophyll contents, which encoded a chlorophyll A-B binding protein. Previously, it was reported that overexpressed a grapevine aldehyde dehydrogenase gene (*ALDH2B8*) in Arabidopsis could improve the leaf chlorophyll contents in the transgenic line under abiotic stresses [63]. Accordingly, we predicted *Glyma15g19670* as a candidate gene, which was from the soybean aldehyde dehydrogenase gene family and was annotated to participate in the chlorophyll catabolic process on SoyBase (Additional file 1: Table S14). Notably, the chlorophyll content was reported to be related to the nitrogen supply in soybean leaves [64]. And we further speculated *Glyma07g15960* from *qCT7Z*, and this gene encoded a nitrogen metabolic regulation protein and was annotated to be associated with the chlorophyll biosynthetic process on SoyBase (Additional file 1: Table S14). Besides, all candidate genes were predicted relatively high gene expression levels in soybean leaf tissues on SoyBase or Phytozome. The six predicted genes should be probed into foresighted functional validations in the future. To sum up, the putative candidate genes may be useful for promoting the research on the hereditary basis of chlorophyll-content traits in soybean leaves.

## Conclusions

In this study, we mapped QTLs of seven soybean leaf chlorophyll-content traits by utilizing a genotyped advanced recombinant inbred line population (ZH, Zhonghuang 24 × Huaxia 3) and its well-constructed high-density genetic map. A total of 78 target traits QTLs were identified and most detected QTLs were novel QTLs for soybean leaf chlorophyll-content traits. Moreover, 13 major QTLs and five important QTL hotspots were classified. Besides, six putative candidate genes were predicted from the hereditary regions of the major QTLs and important QTL hotspots. The identified QTLs and candidate functional genes may directly or indirectly affect the soybean leaf chlorophyll contents in soybean leaves and facilitate future soybean MAS breeding.

## Methods

### Plant materials and field trials

A ZH RIL population with 164 RILs was developed from a cross between ‘ZhongHuang 24’ (ovule parent) and ‘Huaxia 3’ (pollen parent) using a single seed descent (SSD) method derived from individual F_2_ plants [27]. ‘Zhonghuang 24’ is a variety from the Institute of Crop Sciences, Chinese Academy of Agricultural Sciences, which adapts to the Huang-Huai-Hai region. ‘Huaxia 3’ is a high-yield soybean cultivar obtained from South China Agricultural University. The F_12_ ZH RILs were grown together with both parents at 11th March in the spring of 2017 and at 23rd June in the summer of 2017 at Zengcheng experimental station (N23°15′, E113°34′). The detailed climate data were in Table S15 (Additional file 1), including the average temperature and accumulated precipitation of each month. The soil type of the experimental station is sandy loam clay, and the watering regimes were well-watered. A randomized complete block planting with three replications was adopted. Plants were sown in single-row plots, and each plot contained 10 plants per row, with 0.5 m between the rows and 0.1 m between the plants. Field management followed normal soybean production practices for the area.

### Measurements of chlorophyll-content traits and data analyses

According to the detailed description of the different growth stages of soybean by Hanway and Thompson, we chose three representative soybean reproductive growth stages for phenotypic data valuations: the beginning flowering growth stage (R1), the full pod growth stage (R4) and the full seed growth stage (R6) [65]. Due to the diverse growth rates of the RILs, the phenotypic data for the chlorophyll-content traits were collected across different periods. The detailed information for different soybean growth stages was displayed in Table S15 (Additional file 1). The testing and sampling targets were the five plants in the middle of each row.

### Soybean leaf SPAD testing determinations

The soil plant analysis development (SPAD) chlorophyll meter non-destructively measures light transmittance of the leaf in the red and infrared wavelengths at 650 and 940 nm, yielding a numerical output that indicates leaf greenness and chlorophyll concentration [5]. Assessing chlorophyll content in leaf with the SPAD chlorophyll meter is a quick, cheap and effective approach to gain the phenotypic data for soybean leaf chlorophyll-contents. In this study, the SPAD values were scored in the middle leaflets of fully expanded trifoliolate soybean leaves using the SPAD-502 Chlorophyll Meter (Konica Minolta, Japan). Five normal plants in the middle of each row were selected and measured SPAD values of the definite leaves thrice at the top, middle and bottom sites of the plants [30]. The mean testing values of plant different sites were set as the TSP, MSP and BSP, respectively. Moreover, the average SPAD values (ASP) were the means of TSP, MSP and BSP. And all the SPAD values were collected at 9 a.m.

### Soybean leaf pigment extractions and extracted-based chlorophyll evaluations

In this study, we collected the fully developed middle leaflets of the 3rd node on the main stem counting down from the top for soybean leaf pigment extractions at 9 a.m. The leaf samples were from the five single plants of each row. Leaf samples, avoid the main leaf veins, were cut into small pieces and then were ground to crush in liquid nitrogen. About 0.3 g of every sample (*Ws*, the weight of the sample) were sent into a 10 mL centrifuge tube and added 9 mL pre-refrigerated extraction solvent (80% v/v acetone). Samples were incubated at room temperature in the dark for two hours until the crush completely became white. During the incubating process, the centrifuge tubes were preferably shaken up several times to ensure the well-extracted chlorophylls. Then the tubes were centrifuged at 9000×g for 10 min at 25 °C (Centrifuge 5418R, Eppendorf, Hamburg Germany). After centrifuging, 1 mL supernatant liquor of each sample was transferred into an opaque glass vial and diluted the volume to 8 mL by 80% v/v acetone (diluted chlorophyll sample solution). The absorbance of the diluted samples was measured at 645 nm (A645) and 663 nm (A663) using a spectrophotometer (UV-1800 Spectrophotometer, SHIMADZU CORPORATION, Japan), respectively. The chlorophyll contents could be described as follows [52]:

1 $$ \mathrm{Chl}\_\mathrm{A}\ \left(\mathrm{mg}/\mathrm{g}\right)=\frac{\left(12.7\times \mathrm{A}663-2.69\times \mathrm{A}645\right)\times 8\times 9}{1000\times {W}_s}. $$

2 $$ \mathrm{Chl}\_\mathrm{B}\ \left(\mathrm{mg}/\mathrm{g}\right)=\frac{\left(22.9\times \mathrm{A}645-4.68\times \mathrm{A}663\right)\times 8\times 9}{1000\times {W}_s}. $$

### Phenotypic data statistical analyses

Chl_A/B values were obtained from the ratios of chlorophyll a to b. Frequency distribution graphs of chlorophyll-content traits (TSP, MSP, BSP, ASP, Chl_A, Chl_B and Chl_A/B) were depicted by Graphpad Prism 7.0 (http://www.graphpad.com/). Statistical analyses were manipulated with SPSS Statistics 19.0 (https://www.ibm.com/products/spss-statistics) software.

### Genetic map and QTL detection

#### SNP genotyping

Genome-wide SNP genotyping was performed at the Beijing Genome Institute (BGI) Tech, Shenzhen, China. According to the SNP genotyping method of Liu et al. [24], SOAP aligner (http://soap.genomics.org.cn/) was adopted to align the sequencing reads of parents and each RILs to *Glyma*.Wm82. a1. v1. 1 soybean reference genome from Phytozome (https://phytozome.jgi.doe.gov/pz/portal.html) [66]. Using SAMtools (http://samtools.sourceforge.net/) software, the alignments were formatted and converted into realSFS input files for SNP calling and genotyping [67]. By using the sliding window approach with 15 SNPs per window, the genotype for each window and the exchange sites for each individual were determined. Finally, the genotypes of each individual were applied for generating bin information of the high-density genetic linkage map by MSTMap (http://alumni.cs.ucr.edu/~yonghui/mstmap.html) [68]. The Mapchart (https://www.wur.nl/en/show/MapChart-2.32.htm) was used for depicting the genetic linkage maps with the map files from MSTMap [69]. And the genetic linkage map was constructed by the bin markers, which reduced the number of markers into one by integrating the SNPs in the same recombinant block. The bin marker number of each soybean chromosome was presented in Table S4 (Additional file 1). The maximum number of bin markers for each soybean chromosome should be no more than 350.

#### QTL detection

Utilizing the well-constructed genetic linkage map and WinQTLCart2.5 (https://brcwebportal.cos.ncsu.edu/qtlcart/WQTLCart.htm) software, we scanned the QTLs with the composite interval mapping (CIM) method [70, 71]. The logarithms of the odds (LOD) thresholds for QTL significance were determined by a permutation test (1000 replications) with a genome-wide at the 5% level of significance to justify the existence of QTLs. According to the tests, a LOD score of 2.5 was used as a minimum to announce the presence of a QTL in a particular genomic region [72]. Moreover, the location of a QTL was described according to its LOD peak location and the surrounding region with a 95% confidence interval. QTL mapping results were comprehensively compared to SoyBase.

#### QTL naming

The detected QTLs were named according to Cui et al. as follows: initial ‘q’ denotes ‘QTL’; following with chlorophyll-related traits abbreviation letters; the next number is the soybean chromosomes on which the QTL is distributed [73]. Moreover, letters ‘a’ to ‘c’ represent the QTL was detected in the ZH RIL population at the R1, R4 and R6 growth stages in the spring of 2017 at Zengcheng, respectively; letters ‘d’ to ‘f’ represent the QTL was detected in the ZH RIL population at the R1, R4 and R6 growth stages in the summer of 2017 at Zengcheng, respectively; if more than one QTL for a specific trait was dispersed along a certain chromosome, a serial number, viz. -1, 2, etc., is used after the ‘a’ to ‘f’ to describe their order.

### Gene ontology (GO) enrichment analyses and candidate gene predictions

In the current study, the *Glyma*.Wm82. a1. v1. 1 gene model on SoyBase was applied for retrieving the genes that fall into the hereditary blocks of detected QTLs. The AgriGo toolkit v2.0 (http://systemsbiology.cau.edu.cn/agriGOv2) was utilized to perform gene ontology (GO) analyses for these genes [74]. Besides, the GO enrichment terms were filtered (*P* < 0.05 and gene number over five in the selected GO term) and illustrated by TBtools [75]. Furthermore, we looked up these gene annotations on SoyBase (https://www.soybase.org/) and gene tissues expression information on Phytozome and SoyBase. Comprehensively considering the gene annotations and gene tissue expression levels, the candidate genes for chlorophyll-content traits were predicted.

### Candidate gene genomic locations, conserved domains and gene structure depictions

The genomic positions of the candidate genes were stepwise displayed from the anchored bin markers on soybean chromosomes to the candidate genes in the bin markers. The bin markers and the candidate genes were displayed at the ratios based on the soybean genome physical locations. For conserved domains, protein sequences of candidate genes were obtained from Phytozome and set as the inputs to the SMART (http://smart.embl-heidelberg.de/#opennewwindow) online analyzing tool. Gene structures were depicted by TBtools software by using the soybean genome GFF3 file [75]. The graphs were modified by Microsoft Office PowerPoint 2016 and Adobe Illustrator CC 2019.

## Supplementary information

**Additional file 1: Table S1**: The pairwise correlation coefficients between different chlorophyll-content traits in the ZH RILs across environments; **Table S2**: Correlation coefficients between different environments for leaf chlorophyll-content traits in the ZH RILs; **Table S3**: Number of SNPs in the bin markers; **Table S4**: Description of characteristics of 20 chromosomes in the ZH RIL population high-density genetic map; **Table S5**: Segregation distortions of 2639 bin markers among the ZH RIL population; **Table S6**: The 78 QTLs and 70 loci for leaf chlorophyll-content traits in the ZH RIL population across different environments; **Table S7**: Information of Soybase published leaf chlorophyll-content traits QTLs; **Table S8**: Comparisons of the detected chlorophyll-content QTLs with the previously published ones; **Table S9**: Five important chlorophyll-content traits QTL hotspots are associated with several reported yield-related QTLs on SoyBase; **Table S10**: Genes in the hereditary intervals of the 13 major chlorophyll-content trait QTLs; **Table S11**: Genes in the hereditary intervals of the five important QTL hotspots; **Table S12**: Gene Ontology (GO) enrichment analysis of the genes in genetic blocks of the major QTLs and important QTL hotspots; **Table S13**: Filtered GO enrichment terms of the genes in genetic blocks of the major QTLs and important QTL hotspots; **Table S14**: Gene annotations from SoyBase; **Table S15**: Details of field trails, meteorological conditions and soybean growth stages used to determine the soybean leaf chlorophyll-content traits.

**Additional file 2: Fig. S1**: Distribution of the SNP loci throughout 20 soybean chromosomes in the ZH RIL population.

**Additional file 3: Fig. S2**: Schematic and distribution of bin markers on 20 soybean chromosomes in the ZH RIL population.

**Additional file 4: Fig. S3**: Visualization of the filtered GO enrichment terms

## Data Availability

The data sets supporting the results of this study are included in the manuscript. Soybean seeds are available from the Guangdong Subcenter of the National Center for Soybean Improvement, PR China.

## Abbreviations

Aldedh: Aldehyde dehydrogenase
ASP: Average SPAD value
BN: Branch number
bp: Base pair
BSP: Bottom leaves SPAD value
CDS: Coding sequence
Chl_A/B: Ratios of chlorophyll a to b
Chl_A: Chlorophyll a
Chl_B: Chlorophyll b
ChloroA_B-bind: Chlorophyll A-B binding
CIM: Composite interval mapping
cM: Centimorgan
CT: Chlorophyll-content traits
cv.: Cultivar
EP: Effective pod
GBS: Genotyping by sequencing
GO: Gene ontology
GWAS: Genome-wide association study
IP: Invalid pod
kb: Kilobyte
LOD: Logarithms of the odds
MAS: Marker-assisted selective
Mb: Megabyte
MSP: Middle leaves SPAD value
NAD: Nicotinamide adenine dinucleotide
NGS: Next-generation sequencing
NN: Nod number
PAP: Plastid-lipid associated protein
PH: Plant height
QTL: Quantitative trait locus
*R*^*2*^: Phenotypic variance
RAD-seq: Restriction site-associated DNA sequencing
RIL: Recombinant inbred line
SLAF-seq: Specific length amplified fragment sequencing
SNP: Single nucleotide polymorphism
SPAD: Soil plant analysis development
SSD: Single seed descent
SW: Seed weight
TSP: Top leaves SPAD value
UTR: Untranslated region
WGS: Whole-genome sequencing
