# Supplementary figures and images for: QTL mapping for soybean (Glycine max L.) leaf chlorophyll-content traits in a genotyped RIL population by using RAD-seq based high-density linkage map
Source: BMC Genomics. 2020 Oct 23;21:739. doi: 10.1186/s12864-020-07150-4 (PMC7585201; doi:10.1186/s12864-020-07150-4)

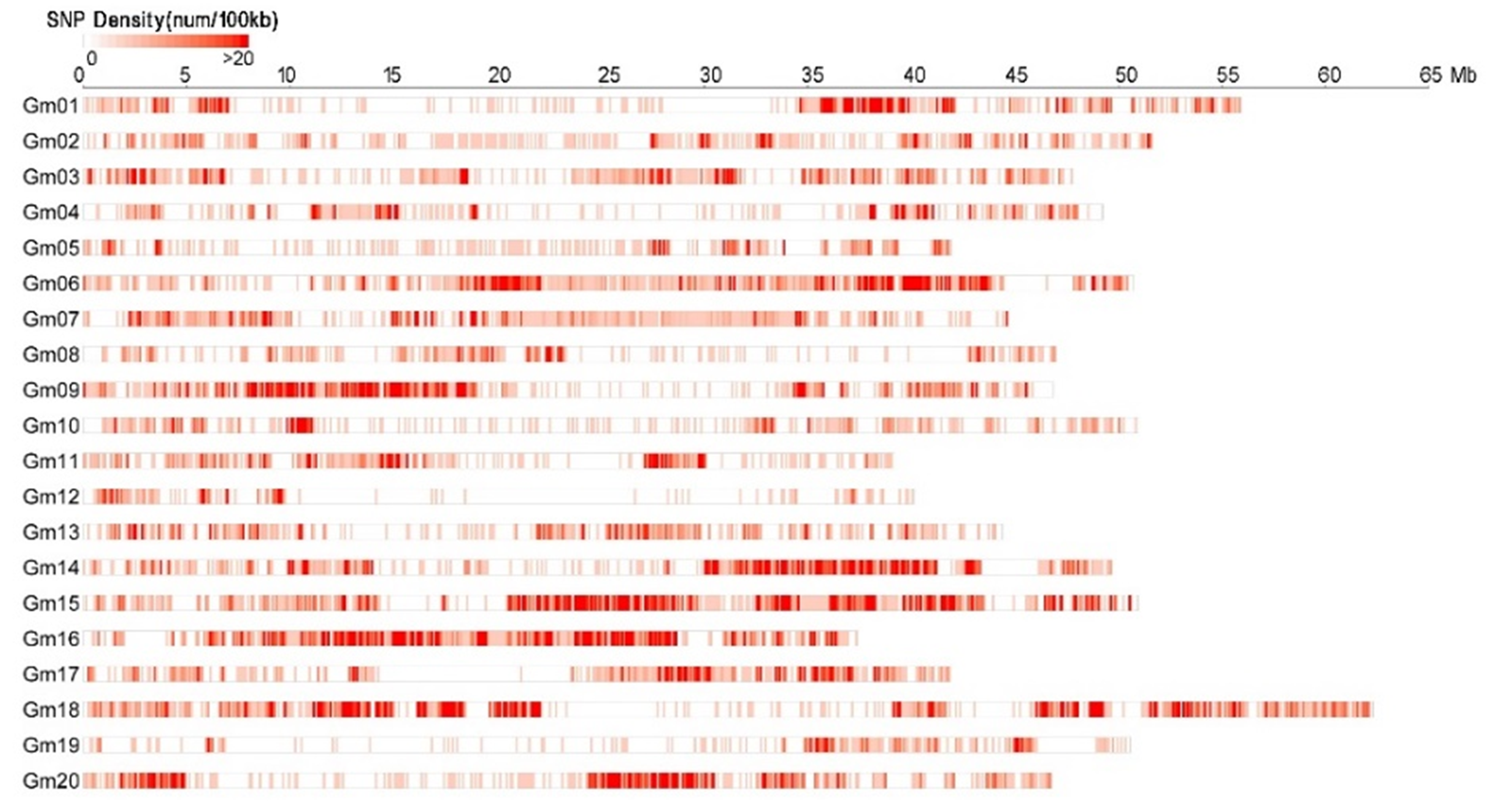

Supplement: Supplementary file 2 — Additional file 2: Fig. S1: Distribution of the SNP loci throughout 20 soybean chromosomes in the ZH RIL population. [file 12864_2020_7150_MOESM2_ESM.tif]

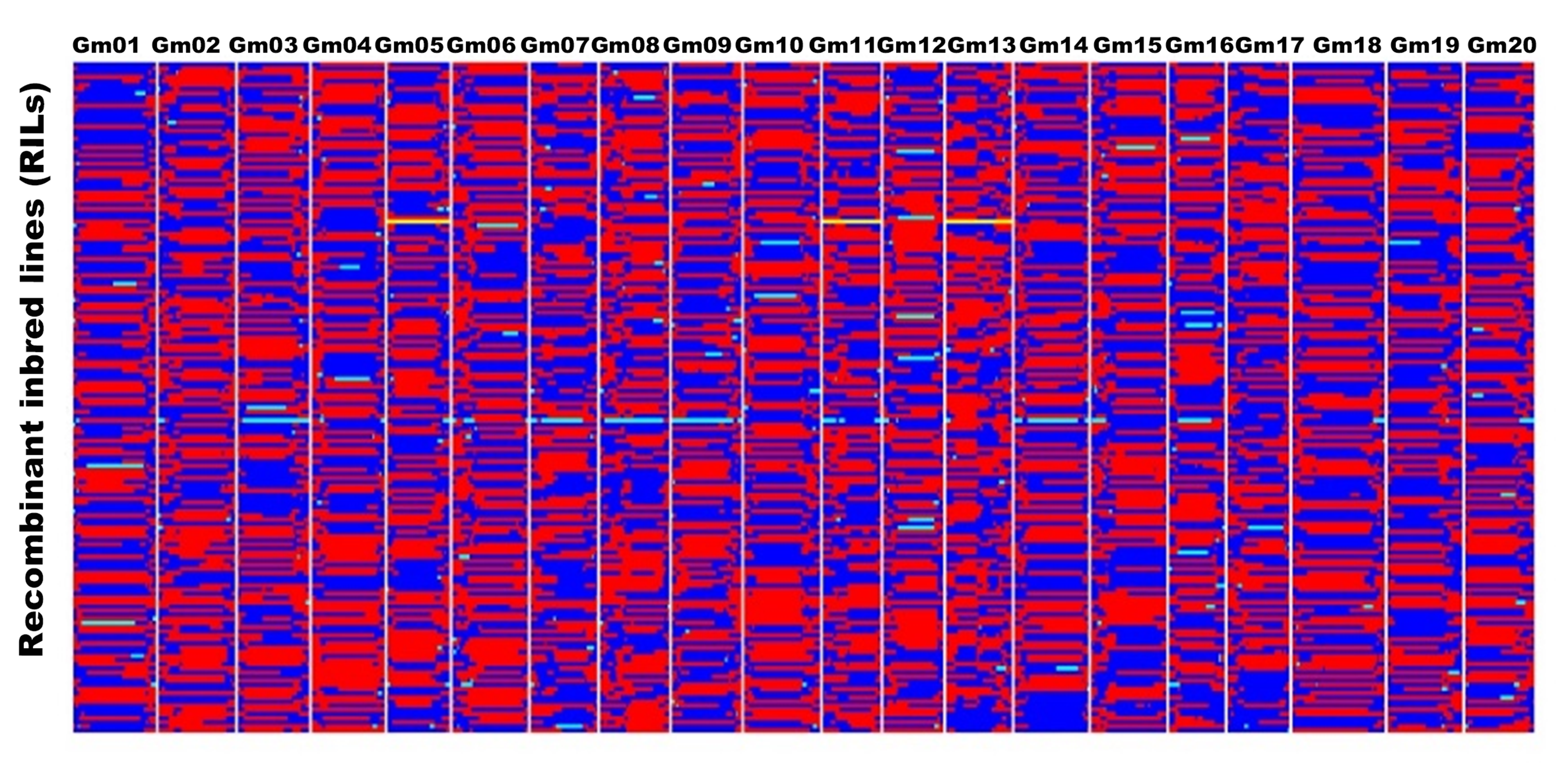

Supplement: Supplementary file 3 — Additional file 3: Fig. S2: Schematic and distribution of bin markers on 20 soybean chromosomes in the ZH RIL population. [file 12864_2020_7150_MOESM3_ESM.tif]

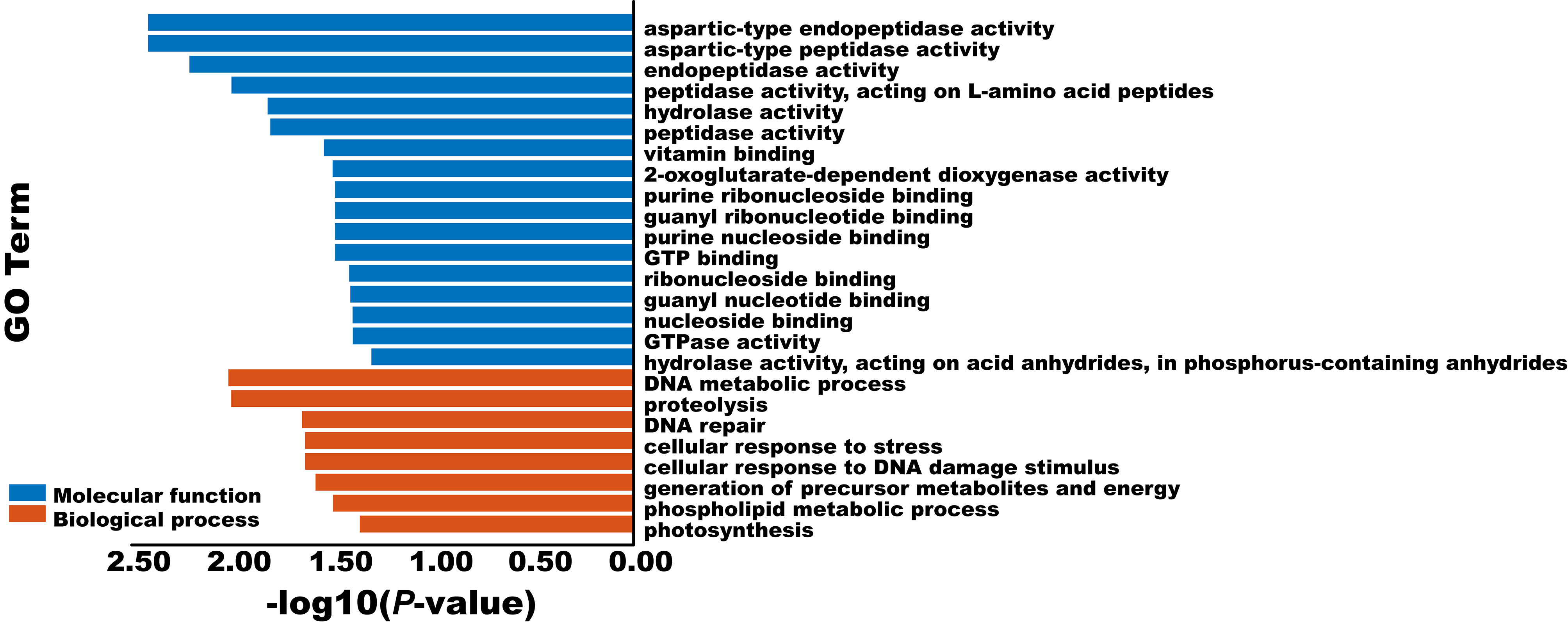

Supplement: Supplementary file 4 — Additional file 4: Fig. S3: Visualization of the filtered GO enrichment terms [file 12864_2020_7150_MOESM4_ESM.tif]
